# Vaccine hesitancy among U.S. parents: a mini review

**DOI:** 10.3389/fpubh.2026.1823951

**Published:** 2026-05-11

**Authors:** Carianne Cornell, Anita Silwal, Ginger Welch, Lucy E. Bailey, Darcy Jones McMaughan

**Affiliations:** 1 School of Community Health Sciences, Counseling and Counseling Psychology, College of Education and Human Sciences, Oklahoma State University, Stillwater, OK, United States; 2 School of Educational Foundations, Leadership and Aviation, College of Education and Human Sciences, Oklahoma State University, Stillwater, OK, United States

**Keywords:** childhood immunization, vaccine, vaccine adverse reaction, vaccine hesitancy, vaccine injury

## Abstract

Parental vaccine hesitancy in the United States continues to shape childhood immunization patterns and population-level disease prevention. Although extensive evidence demonstrates that vaccines are safe and effective, many parents base vaccination decisions on concerns about adverse reactions that extend beyond risk estimates derived from epidemiological data. Parents construct these interpretations through personal experiences with adverse reactions as well as through stories circulating within families, social networks, media, and online environments. This narrative mini review synthesizes interdisciplinary literature on parental vaccine hesitancy in the United States, focusing on how perceived and experienced adverse reactions influence attitudes toward childhood vaccination. The review examines contextual factors associated with hesitancy, including historical events, healthcare experiences, and interpretations of vaccine safety. By integrating public health research with parental vaccine perspectives, this review identifies gaps between population-level safety evidence and individual risk perception. Addressing these gaps offers opportunities to strengthen vaccine confidence, enhance public health communication, and support informed parental decision-making without exacerbating mistrust or polarization.

## 1 Introduction to vaccine hesitancy

**Figure 1:**
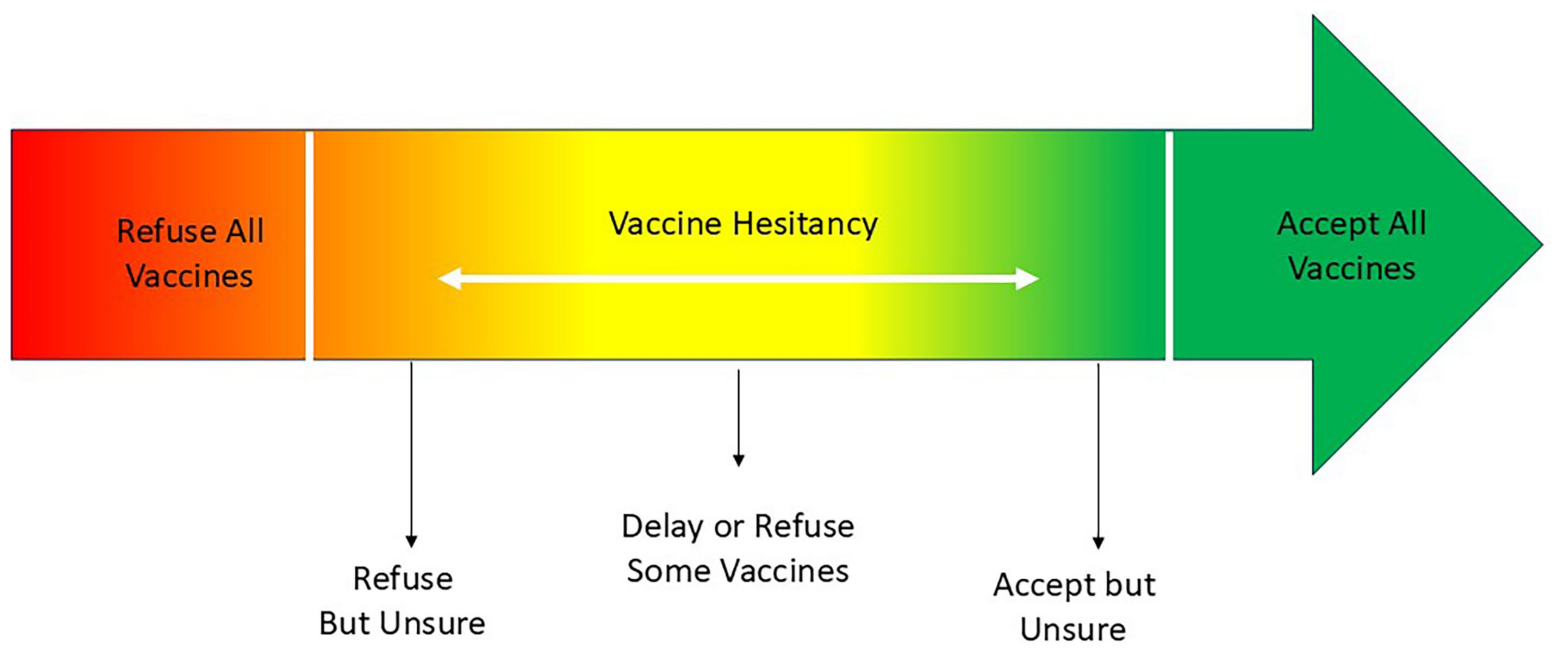
Continuum of vaccine acceptance [adapted from MacDonald (52)].

Public hesitation and suspicion surrounding vaccination have been documented since its introduction; however, researchers introduced the term *vaccine hesitancy* in the early 2010s to capture this phenomenon more precisely. Opel et al. (1) conceptualized vaccine hesitancy as a spectrum, with complete vaccine refusal at one end and complete acceptance at the other. Within this framework, vaccine-hesitant individuals occupy positions along the continuum without aligning fully with either polarized stance (1–3) (Figure 1).

Olson et al. (4) synthesized the literature and defined vaccine hesitancy as uncertainty or lack of confidence in vaccination that results in delaying or refusing immunization. The World Health Organization (WHO) defined vaccine hesitancy as a delay in acceptance or refusal of vaccines despite their availability and characterizes it as a complex, context-specific phenomenon that varies across time, place, and vaccine type (5). Vaccine hesitancy holds three interrelated dimensions—cognitive and affective processes (how people think and feel about vaccines), behavioral responses (actions including delaying, selectively accepting, or refusing vaccines), and decision-making dynamics (negotiating risk and trust of vaccinations)—emphasizing hesitancy as a state of indecisiveness surrounding vaccination decisions (6). Individual, peer and contextual factors may influence hesitancy, including perceptions that vaccines are unnecessary, inconvenient, or sufficiently trustworthy (4, 7–9). Although definitions vary, embedded within these definitions is the concept of vaccine confidence, which refers to trust in vaccines and the systems that deliver them, a sense of control over health decisions, satisfaction with available knowledge, and confidence in a positive health outcome (10).

Understanding vaccine behaviors in the United States requires some understanding of the American health care system. The United States health care system is a complex mix of public and private financing, involving both for-profit and non-profit entities (11). In the absence of universal health coverage, many Americans rely on employer-sponsored health insurance, as well as government programs including Medicare and Medicaid, which serve older adults and low-income families (11, 12). This fragmented structure contributes to significant variability in access to care, including vaccination, quality of care, and health outcomes across populations and geographic regions (13). However, both public and private sources have worked to make vaccines more available through the Vaccines for Children (VFC) program (14).

To protect public health, legal responses to anti-vaccination sentiment in the United States have primarily focused on immunization mandates for public school attendance and restrictions on non-medical vaccine exemptions. Such mandates have been consistently upheld by the U.S. Supreme Court [e.g., (15)], with subsequent rulings continuing to affirm the constitutionality of vaccine requirements. More recent state-level policy changes in California and New York, which implemented stricter exemption criteria, further illustrate legal efforts in response to declining vaccination coverage. Alongside these legal approaches, interventions within clinical settings have been implemented to address vaccine hesitancy, including provider communication strategies, vaccine counseling, and efforts to reduce missed opportunities for vaccination (2, 16). However, some evidence suggests that vaccine mandates and certain policy approaches may be perceived as overly restrictive, and that communication strategies emphasizing high-pressure messaging may contribute to increased distrust and hesitancy among parents (2, 17–19).

## 2 History of vaccine hesitancy

Since the introduction of the first smallpox vaccine in the 1796, suspicions about vaccinations have existed globally (8, 20). In the United States (U.S.), the smallpox outbreak of 1902 in Cambridge, Massachusetts led the city health board to mandate adult vaccination. Following this mandate, in 1905, the landmark case *Jacobson v Massachusetts* challenged the state’s authority to limit personal freedom for reasons regarding public health (8). While this case led to the Supreme Court ruling that such state restrictions of individual liberty are a justified action when great dangers to public safety are present (8), later vaccine rollouts, safety incidents, and the establishment of surveillance and compensations systems significantly influenced public views on vaccinations.

Throughout the mid-20th century, new vaccines were introduced to prevent illness including tuberculosis, yellow fever, whooping cough, tetanus, and polio. These vaccinations substantially reduced child mortality rates, including a 70% improvement in infant mortality (21). Furthermore, public acceptance of vaccines increased when polio cases declined after the Salk vaccine was available to the public (8). Vaccine acceptance increased through witnessing the decrease in polio’s devastating outcomes such as paralysis or death among children. However, a significant vaccine production error that followed once again shifted public opinions. In 1955, several lots of the Salk vaccine were released that contained active polio virus instead of the usual inactivated polio virus (22). Although required safety testing procedures were completed, the production error resulted in approximately 120,000 children receiving vaccines containing live virus leading to an estimated 70,000 cases of mild polio, 200 cases of permanent paralysis, and 10 deaths (8). This incident is often cited as an early foundation for public distrust in the pharmaceutical industry (8); at the same time, it prompted significant improvements in vaccine manufacturing and regulatory protocols (22). Shortly after the Salk vaccine error, contaminated polio vaccines were released in the U.S. The contamination included SV40, a virus found in monkey kidney cell cultures used to create the vaccines. Approximately 10–30% of U.S. polio vaccines were contaminated with the SV40 virus, which was feared to cause certain cancers. Although no direct link was determined and currently all polio vaccines administered are free of SV40, this incident contributed to public apprehension toward vaccines (22).

In 1976, public health officials launched a swine flu vaccination campaign in preparation for a potential influenza pandemic. After more than 45 million doses were administered, surveillance identified a small increased risk of Guillain-Barre Syndrome (GBS), estimated at approximately one additional case per 100,000 vaccinations (23). Although subsequent research has consistently demonstrated that GBS following influenza vaccination is rare (24) and the risk of GBS is higher following influenza infection (8), early safety concerns shaped public discourse. A report conducted by the Institute of Medicine later concluded that available evidence was insufficient to confirm or reject a causal relationship between influenza vaccination and GBS (19). Suspected associations between flu vaccination and GBS continue to amplify safety concerns and contribute to vaccine hesitancy (19).

Following controversies in 1974 and 1982 that linked the whole-cell diphtheria, tetanus, and pertussis (DTP) vaccine to concerns about severe neurological complications in children, public confidence in vaccine safety declined again and parental activism intensified (25, 26). Concerned parents formed the Association of Parents of Vaccine-Damaged Children (APVDC), which became a central force advocating for recognition of vaccine injuries and the establishment of compensation mechanisms for affected families (26). In parallel, the National Childhood Encephalopathy Study (NCES) was commissioned by the Joint Committee on Vaccination and Immunization (JCVI), a federal government institution, to systematically investigate reports of serious neurological illness following DTP vaccination.

The NCES employed a case–control design and followed infants and toddlers aged 2 to 35 months who were admitted to hospitals with specific acute neurological diagnoses (27). Although the study reported a statistically significant temporal association between DTP vaccination and serious neurological illness, such cases were rare and complete recovery was the most common outcome (28). Despite these findings, public confidence in vaccine safety declined substantially during this period, contributing to a marked reduction in DTP vaccination coverage.

In the mid-1980s, public opinion again began to shift following the release of the NBC documentary *DPT: Vaccine Roulette* (29) and the book *A Shot in the Dark* (30), both of which claimed that children experienced seizures and permanent neurological damage following DTP vaccination. Although subsequent scientific research did not substantiate these claims, vaccine uptake declined and litigation against vaccine manufacturers increased (8). In response, the U.S. Department of Health and Human Services established the Vaccine Adverse Event Reporting System (VAERS) as part of the National Childhood Vaccine Injury Act of 1986, along with the National Vaccine Injury Compensation Program (NVICP). Collectively, these mechanisms were designed to stabilize the vaccine market by shifting liability away from vaccine manufacturers and toward a no-fault, federal government-administered compensation system (31).

Additionally, these efforts were justified through principles of fairness and solidarity, whereby individuals who experience rare but serious adverse events following vaccination are compensated by society as a whole, providing compensation through a trust fund financed by excise tax imposed on each dose of vaccine (32, 33). These policies and compensation allow for the acknowledgment that vaccination serves a collective public health good and that the burdens of this collective benefit should not fall disproportionately on families affected by adverse events (32).

While these policies were intended to protect manufacturers and promote vaccine compliance, vaccine injury compensation systems may unintentionally reinforce vaccine hesitancy. Research indicates that the difficulty of proving a causal link between vaccination and alleged injury is a persistent limitation of compensation programs (32, 34). Many claims are dismissed or result in no compensation due to stringent evidentiary standards, procedural barriers, or reliance on tort-based legal principles, which can leave families feeling unsupported or disbelieved (31, 32, 34). Mustaffa et al. (34) further note that lengthy timelines, financial costs, filing deadlines, and complex legislative procedures can impede timely and equitable compensation. While the absence of definitive causal determination does not necessarily diminish vaccine hesitancy; these structural and procedural challenges may exacerbate it by fostering perceptions that adverse experiences are minimized or inadequately addressed. Attitudinal factors also shape the perceived legitimacy of compensation programs, as some members of the public question whether governments should assume responsibility for harms associated with vaccine injury, further impacting trust in vaccination systems (31).

The 1990’s also brought concerns regarding thiomersal in some vaccines. Thiomersal, a preservative used in certain vaccines to prevent germ growth that may lead to severe reactions among recipients (35), contains mercury, a heavy metal commonly known to cause severe health effects due to organic mercury poisoning. CDC findings show no evidence of harm caused by thimerosal in vaccines and it has a long record of safe use in medical products (83). However, for precautionary reasons, the Public Health Service, the American Academy of Pediatrics, and vaccine manufactures agreed to reduce or eliminate thiomersal from vaccines and encouraged doctors to postpone the birth dose of the Hepatitis B vaccine to children without risk of contracting the disease (8, 36). By 2001, thiomersal was removed from all childhood vaccines (35). These actions led to confusion among medical professionals and fear among parents, assuming thiomersal was harmful therefore undermining trust surrounding vaccine regulation. Parents formed advocacy groups that credited thiomersal for their children’s autism. The resulting thiomersal controversy was leveraged for political purposes when Governor Arnold Swarzenegger banned thiomersal-containing flu vaccines, with five other states following suit (2, 8). Such social influences compounded the issue of lost trust in vaccine safety and regulation.

Although thiomersal has been removed from routine childhood vaccines, other ingredients, particularly adjuvants, play a role in vaccine hesitancy (37). Aluminum salts were the first licensed adjuvants and remained the only option for decades (38). Since the 1990’s, additional adjuvants including MF59, AS01, AS04, AS03, and cytosine phosphoguanosine have been introduced to enhance immune response and improve vaccine effectiveness (39). While researchers are still investigating their precise molecular mechanisms, clinical trials and post-licensure surveillance have evaluated their safety (39). Adjuvants are generally well tolerated, though they may increase short-term local and systemic reactions, and rare adverse events are continually monitored. Some scholars have raised concerns about potential effects related to the immune system, including Autoimmune/Inflammatory Syndrome Induces by Adjuvants (ASIA) (40). While the Food and Drug Administration (FDA) requires a rigorous and extensive process to determine vaccine safety and effectiveness, adjuvants are assessed as components of vaccine formulations rather than standalone products (41). Some vaccine-hesitant parents raise concerns that licensed adjuvants expanded after establishing liability protections for manufacturers particularly amid ongoing scientific investigation into their mechanisms of action. At the same time, extensive surveillance data demonstrate that serious adverse reactions remain rare, and vaccines continue to substantially reduce morbidity and mortality (40).

Vaccine controversy continued in 1998 when Andrew Wakefield published a report in *The Lancet* claiming the Measles, Mumps, Rubella (MMR) vaccine caused autism [(42); RETRACTED]. Wakefield’s research hypothesized the MMR vaccine may cause intestinal inflammation and neurological dysfunction that could potentially lead to autism, and suggested single vaccines administered further apart may be an appropriate protocol [(42); RETRACTED]. Although Wakefield denies accusations of fraud and misconduct, the paper was retracted by *The Lancet* in 2010 because of several inconsistencies including carefully chosen subjects and funding that produced explicit conflict of interests (43). Numerous subsequent studies found no link between the MMR vaccine and autism, yet Wakefield’s research is credited for leading to serious public health consequences including decreased MMR vaccine utilization and measles outbreaks in California and Minnesota over the past decade (8) with the most recent outbreak in 2025.

The most recent social influence on vaccine hesitancy is the global pandemic of Covid-19. Between 2019 and 2022 U.S. citizens experienced social distancing, lockdowns, and over one million deaths due to Covid-19 (23). Due to years of foundational research on mRNA platforms and coronaviruses prior to the pandemic, Covid-19 vaccines were able to be developed and administered within approximately one year after the virus emerged (44). Researchers estimate that Covid-19 vaccines have prevented millions of deaths, hospitalizations and infections due to the virus (45). Despite its success, approximately one third of the U.S. population either declined or felt uncertain about the Covid-19 vaccine (46). The rapid rollout of COVID-19 vaccines, combined with the widespread circulation of misinformation on social media, shaped public confidence, risk assessment, and perceptions of collective responsibility toward COVID-19 vaccination (47).

## 3 Parents and vaccine hesitancy

In recent years, research indicates a noticeable increase in U.S. parents who choose to deviate from the CDC recommended childhood immunization schedule, including delaying, partially vaccinating, or refusing components of the schedule (48). Additionally, national survey data indicate that many parents report limited confidence in the safety of the recommended vaccine schedule, a pattern associated with increased non-adherence to the CDC schedule (49). Approximately 75% of U.S. parents follow the recommended childhood vaccination schedule, receiving all vaccinations at age-appropriate times (50), leaving roughly 25% of U.S. children under-vaccinated. National CDC data indicate that vaccine hesitancy among U.S. parents has increased in recent years, particularly among parents of children aged 5–11 years. Data from the CDC’s National Immunization Survey show that approximately one in five children has a parent who expresses vaccine hesitancy, while parental vaccine hesitancy among children aged 5–11 years increased from 19.8% in 2019 to 21% in 2022 (51).

More recent indicators align with this trend. CDC reports show that kindergarten vaccination coverage declined in more than half of U.S. states during the 2024–2025 school year, with rates of MMR, DTap, and Polio vaccination dropping from above 95%, which is the optimal coverage needed for herd immunity, to approximately 92% (66). Concurrently, the proportion of kindergarteners with vaccine exemptions increased nationwide from 3.3 to 3.6%, with 17 states reporting exemption rates of 5% or higher, representing approximately 138,000 children exempt from one or more required vaccinations (66). Together, these patterns underscore opportunities to strengthen routine childhood vaccine uptake in the United States.

Fear of adverse reaction is among the most consistently identified drivers of parental vaccine hesitancy. Multiple studies have found the perceived risk of side effects, both mild and severe, is strongly associated with lower intentions to vaccinate despite significant evidence indicating serious adverse events are rare (2, 17). Parents often view what are typical vaccine reactions, such as low-grade fever, as alarming; they worry about harming their child’s immune system with an “overload” of vaccinations (52).

Decisions surrounding child vaccine uptake are influenced by multiple factors including care-taker’s experiences within medical settings, others’ experiences within the medical setting, relationship with the medical provider, and feelings toward the overarching medical system (53). In current literature, past experiences may involve personal history of adverse vaccine reaction or hearing detailed third-party accounts of previous adverse vaccine reaction (48, 54, 55). Lived experiences may impact vaccine decision making. Negative vaccine experiences may impact caregivers’ vaccination decisions with parents experiencing a strong emotional response due to previous vaccine-related traumatic events within the medical field (53). Previous research indicates personal knowledge of a fellow parent whose child experienced a severe adverse event due to vaccination is associated with increased vaccine hesitancy (48, 56). While personal experiences with one’s own child lacks significant research, initial analysis finds parents who decline or delay vaccinations were more likely to report a previous adverse reaction, either severe or minor (48). Furthermore, parents who believe their child’s autism spectrum disorder is due to an adverse vaccine reaction are more likely to delay or decline vaccines for the younger siblings (57).

Childhood vaccination is widely promoted as a cornerstone of public health; however, a measurable proportion of parents report adverse reactions following their child’s immunizations. Survey data find 30–40% of parents recall at least one adverse vaccine reaction among their children (58, 59). It is estimated that vaccine safety surveillance systems capture approximately 13% of adverse events (60), indicating reports of adverse vaccine reaction occur outside of vaccine safety surveillance systems.

Within clinical settings, mothers who report adverse events following vaccination frequently describe feeling dismissed, disbelieved, or marginalized by providers, leading to intensified distress and negative influence on long-term perceptions of vaccination. Research suggests that clinicians may attribute reported symptoms to coincidence, anxiety, or unrelated developmental processes without engaging empathetically with parents’ observations (61, 62). Mothers have reported that clinicians view their experiential knowledge as caregivers as less credible than professional expertise, particularly when symptoms are atypical, delayed, or difficult to categorize (63). For example, some mothers describing developmental regression following vaccination report being reassured that “correlation does not imply causation.” While scientifically accurate, such responses may unintentionally curtail dialogue and leave mothers feeling unheard rather than informed.

Beyond clinical settings, mothers who publicly share vaccine injury narratives may experience social stigma, including being labeled an “anti-vaccine,” “misinformed,” or “irresponsible,” regardless of whether they support vaccination in general (31). Such framing can suppress nuanced discussions and discourage caregivers and patients from sharing experiences that deviate from dominant public health narratives. Online parenting forums or social media platforms have been reported to remove, flag, or provoke hostile responses to posts describing adverse vaccine reactions, reinforcing the perception that certain experiences are unacceptable to discuss publicly (64). Historically, this marginalization is also reflected in institutional contexts; a 2004 *Los Angeles Times* report detailed the adversarial nature of vaccine injury compensation proceedings, describing a family’s experience navigating skepticism within legal and medical systems (65).

It is important to note that existing research does not suggest healthcare providers or institutions intentionally seek to dismiss parental narratives. Rather, these experiences reflect broader structural tensions between population-level evidence, risk communication practices, and individual lived experiences.

## 4 Impact and recommendations

As the presence of parental vaccine hesitancy trends upward, we observe the occurrence of certain vaccine preventable diseases (VPDs) among children do the same. Pertussis, or whooping cough cases in the U.S. increased from 7,063 cases in 2023 to 35,435 cases in 2024 with preliminary case reports for 2025 remaining elevated (66). The U.S. measles outbreak of 2025 involved thirty-four states reporting a total of 1,168 confirmed measles cases, disproportionately affecting young children (82). Nearly 30% of cases occurred in children under age 5-a group particularly vulnerable to severe complications (82). During the outbreak, 85 children were hospitalized, highlighting the significant strain on healthcare resources, and 2 children died, emphasizing the potentially deadly nature of measles even in current times.

In the age of social media, many people actively seek vaccine information through content on a variety of platforms (67, 68). Social media is well-documented as an avenue for the dissemination of inaccurate information, particularly impacting reluctancy toward vaccines (67, 69). While information gained from traditional mass media sources decreases vaccine skepticism, the use of social media for obtaining health information counteracts this impact (67, 70). Despite vaccine hesitations and the influence of social media, physicians and other healthcare providers are still considered highly trusted resources for medical advice, including advice on vaccinations (71). Research consistently finds that health care providers play a significant role in vaccine decision making because they are the primary source to whom care takers turn to for vaccine information (56, 72–74). Aspects of positive relationships with healthcare providers that lead to vaccine acceptance include trust, communication, and respect (75, 76).

The role of healthcare providers proves to hold such heavy influence that communication strategies have been developed, providing recommendations that foster adequate trust, communication, and respect with vaccine hesitant parents. Piccoliori et al. (77) developed a research-based guide for practitioners with the following communication recommendations: active listening, asking open-ended questions, providing information, showing empathy, motivational interviewing, and avoiding persuasion. Vaccine safety is consistently a top concern among vaccine hesitant parents (78–80), creating a critical need among healthcare professionals to both understand vaccine safety and how to communicate vaccine safety to parents (79).

**Figure 2:**
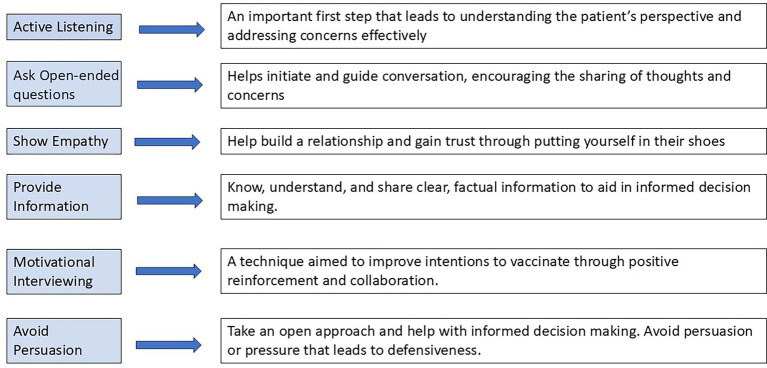
Communication attitudes when interacting with vaccine hesitant parents [adapted from Piccoliori et al. (77)].

While the impact of vaccine hesitancy is evident in decreases in vaccine uptake and the resurgence of VPDs, the fear of adverse reaction and marginalization of shared experiences involving adverse reaction remain important to consider. Recognizing and engaging with parental narratives does not require abandoning scientific rigor, but evidence suggests that attentive listening and validation may strengthen trust, communication, and shared decision-making between providers and families (7, 81). Strategies involving healthcare providers and adequate communication, such as the guide developed by Piccoliori et al. (77) (Figure 2), may transcend into other influential areas including social media, policy environments and legal systems, where marginalization appears to amplify hesitancy and decrease trust. When considered collectively, the evidence presented in this review suggests that trust in vaccination decisions is not only shaped by scientific information but also by how individuals perceive the quality, tone, and inclusiveness of communication throughout clinical and public domains. These dynamics illustrate important considerations for future public health efforts aimed at understanding and addressing vaccine-related attitudes and behaviors among parents.
